# Development of an *in vitro* Assay, Based on the BioFilm Ring Test^®^, for Rapid Profiling of Biofilm-Growing Bacteria

**DOI:** 10.3389/fmicb.2016.01429

**Published:** 2016-09-21

**Authors:** Enea G. Di Domenico, Luigi Toma, Christian Provot, Fiorentina Ascenzioni, Isabella Sperduti, Grazia Prignano, Maria T. Gallo, Fulvia Pimpinelli, Valentina Bordignon, Thierry Bernardi, Fabrizio Ensoli

**Affiliations:** ^1^Clinical Pathology and Microbiology Department, San Gallicano Institute, Istituti di Ricovero e Cura a Carattere ScientificoRome, Italy; ^2^Infectious Disease Consultant, San Gallicano Institute, Istituto di Ricovero e Cura a Carattere Scientifico (IRCCS)Rome, Italy; ^3^BioFilm Control, Biopole Clermont LimagneSaint Beauzire, France; ^4^Department of Biology and Biotechnology C. Darwin, Sapienza University of RomeRome, Italy; ^5^Biostatistics, San Gallicano Institute, Istituto di Ricovero e Cura a Carattere Scientifico (IRCCS)Rome, Italy

**Keywords:** biofilm, BioFilm Ring Test, crystal violet, *Pseudomonas aeruginosa*, *Klebsiella pneumonia*, *Staphylococcus aureus*, *Staphylococcus epidermidis*, *Ralstonia mannitolilytica*

## Abstract

Microbial biofilm represents a major virulence factor associated with chronic and recurrent infections. Pathogenic bacteria embedded in biofilms are highly resistant to environmental and chemical agents, including antibiotics and therefore difficult to eradicate. Thus, reliable tests to assess biofilm formation by bacterial strains as well as the impact of chemicals or antibiotics on biofilm formation represent desirable tools for a most effective therapeutic management and microbiological risk control. Current methods to evaluate biofilm formation are usually time-consuming, costly, and hardly applicable in the clinical setting. The aim of the present study was to develop and assess a simple and reliable *in vitro* procedure for the characterization of biofilm-producing bacterial strains for future clinical applications based on the BioFilm Ring Test® (BRT) technology. The procedure developed for clinical testing (cBRT) can provide an accurate and timely (5 h) measurement of biofilm formation for the most common pathogenic bacteria seen in clinical practice. The results gathered by the cBRT assay were in agreement with the traditional crystal violet (CV) staining test, according to the κ coefficient test (κ = 0.623). However, the cBRT assay showed higher levels of specificity (92.2%) and accuracy (88.1%) as compared to CV. The results indicate that this procedure offers an easy, rapid and robust assay to test microbial biofilm and a promising tool for clinical microbiology.

## Introduction

Biofilm formation is a key property of microbial cells, which allows long-term survival both in natural ecosystems and animal hosts (Høiby et al., 2010a). Bacteria growing in a biofilm matrix are intrinsically more resistant to environmental agents and have been shown to tolerate antibiotic concentrations 10 to 1000-fold higher than the corresponding planktonic counterpart (Hill et al., 2005). Both Gram-positive and Gram-negative bacteria are capable of forming biofilm (Donlan, 2001). In fact, microbial biofilm represents a serious problem in industry and medicine, including surgery, and dentistry. Being responsible of high microbial adherence, invasiveness and persistence, microbial biofilm represents a major causative agent of chronic and recurrent infections (Lindsay and von Holy, 2006). More than 80% of human infectious diseases are biofilm-related, having a significant impact on patient morbidity and healthcare costs (Römling and Balsalobre, 2012). The formation of biofilms on implantable medical devices causes persistent infections, which account for more than 60% of reported nosocomial infections (Darouiche, 2004). In this setting, catheter-related infections are the most serious and costly adverse events caused by biofilm-producing bacteria, frequently resulting in treatment failure and requiring the removal of the device (Costerton et al., 2003). Typical examples of biofilm-associated diseases include “difficult infections,” such as bone and joint infections, caused by *Staphylococcus aureus* (Hackett et al., 2015; Jacqueline and Caillon, 2014; Gbejuade et al., 2015), infective endocarditis, mainly caused by staphylococci or streptococci, which are associated with high mortality rates (Furuya and Lowy, 2003; Suetens et al., 2007; Otto, 2009) as well as chronic pulmonary infections and respiratory failure caused by *Klebsiella pneumoniae* (Hennequin and Robin, 2016) and *Pseudomonas aeruginosa* (Høiby et al., 2010b; Ciofu et al., 2015).

Antibiotic treatment, either empirical or based on drug resistance profiling, is often poorly effective against biofilm-producing bacterial cells (Høiby et al., 2010a; Ciofu et al., 2015). In fact, in addition to the production of the biofilm extracellular matrix, biofilm-embedded cells differ from the planktonic counterparts for other properties including a reduced growth rate and a distinct gene expression (Beloin and Ghigo, 2005). The latter is due to the activation of complex mechanisms of gene signaling involving 40–60% of the prokaryotic genome (Beloin and Ghigo, 2005). These mechanisms, which allow biofilm-producing bacteria to adapt against environmental stress conditions, are also responsible for an increased tolerance to antimicrobials (Beloin and Ghigo, 2005; Percival et al., 2015). However, the antibiotic–resistance profiles are traditionally performed *in vitro* on growing planktonic cells and do not take into account the impact of biofilm production by microbial cells. Thus, the resulting antibiotic susceptibility profile might not be representative of the bacterial drug susceptibility/resistance *in vivo* (Van Acker et al., 2014; Olsen, 2015). Laboratory assays capable of evaluating biofilm production and the susceptibility of biofilm-forming microorganism to antimicrobial drugs still represent unmet needs in clinical microbiology.

An ideal diagnostic technique should be low cost, reliable and capable of providing a timely characterization of biofilm production by the microorganism(s) to be easily incorporated into routine clinical laboratory testing.

A variety of quantitative methods, either direct or indirect, have been developed (Peeters et al., 2008), either based on colorimetric (Stepanovic et al., 2000; Joseph and Wright, 2004) or microscopic techniques (Benoit et al., 2010; Müsken et al., 2010). At present, the crystal violet (CV) staining is the most widely used method for *in vitro* biofilm quantification, due to its relative simplicity and sensitivity (Christensen et al., 1985; Stepanovic et al., 2000). This method, however, presents important limitations. In fact, it usually requires at least 24/48 h of incubation and repeated processing steps, which lead to large standard deviation of the readouts, making the method neither easily feasible for standardization nor adaptable to large-scale screening. Recently, a new technology, namely the BioFilm Ring Test® (BRT), has been proposed for the assessment of bacterial biofilm. The principle is based on the immobilization of magnetic beads by the growing biofilm matrix *in vitro* (Chavant et al., 2007). The BRT method is simple and does not require extensive handling (i.e., does not require repeated washing and staining steps), thus allowing for the standardization of the procedure, a necessary prerequisite to ensure clear and reproducible readouts (Olivares et al., 2015). However, although having a great potential, the original procedure was not capable of providing, in a single determination, direct information about the “dynamic” and “strength” of biofilm production by different microorganisms. Further, it required repeated measurements, to be performed at different time points, to estimate the formation of microbial biofilm, posing a further, important limitation for use in the clinical setting.

The aim of this study was to develop a simple protocol to evaluate bacterial biofilm production based on the BRT technology, for high throughput screening for future applications in clinical microbiology.

The procedure relies on the measurement of biofilm formation at an early stage. The concept is that, within a given time period (i.e., 5 h), the fewer initial concentrations of bacterial cells that inhibit the aggregation of the microparticles, the stronger is their ability to produce biofilm. Conversely, if high bacterial concentrations are not able to prevent the aggregation of the microparticles they are considered low biofilm producers. To test this concept, the biofilm forming ability of both Gram-positive and Gram-negative clinical isolates was measured and compared to that of laboratory strains with known biofilm phenotype used as internal reference, to ensure accuracy and reproducibility of the results. Inter-assay reproducibility was determined by comparison with the CV assay.

## Materials and methods

The Central Ethics Committee I.R.C.C.S. Lazio, section of the Istituti Fisioterapici Ospitalieri in Rome, approved this study (Prot. CE/1016/15–15 December 2015, trials registry N. 730/15).

### Strains and growth conditions

Reference strains were from American Type Culture Collection (ATCC): *S. aureus* ATCC 25923 (Sa25923) and ATCC 6538 (Sa6538); *Staphylococcus epidermidis* ATCC 14990 (Se14990) and ATCC 12228 (Se12228); *K. pneumoniae* ATCC 700603 (Kp700603) and ATCC 13883 (Kp13883); *P. aeruginosa* ATCC 47085 (Pa47085), ATCC 9027 (Pa9027), and Pa14 (Pa14); *Ralstonia mannitolilytica* LMG 6866 (LMG6866) and BK931 (BK931). Bacteria were cultured aerobically at 37°C; culture media were blood agar, chocolate agar or McConkey agar (Oxoid, Hampshire, UK).

### Bacterial panel selection

A total of 52 clinical isolates collected from patients with nosocomial infections admitted at the IFO Hospital (Rome, It) were assessed. Bacteria were collected from different materials including ulcer infections, intravenous, and urinary catheter tips, blood, urine, sputum, and nasobronchial lavage specimens. An ulcer was classified as chronic if it existed from at least 3 months (Dissemond, 2006). The study also included strains showing increased resistance to commonly used antibiotics as determined by standard Antibiotic Susceptibility Test by the VITEK2 system (bioMérieux, Florence, Italy). In particular, *P. aeruginosa* isolates resistant to three or more classes of antibiotics were considered multidrug drug resistant organisms (MDR) (Magiorakos et al., 2012). *K. pneumoniae* resistant to most beta-lactam antibiotics, including penicillins, cephalosporins, and the monobactam aztreonam and growing on selective chromogenic medium chromID ESBL (bioMérieux, Florence, Italy) were classified as extended spectrum beta-lactamase (ESBL). Phenotypic detection of ESBL producers was further defined using disk approximation tests (Oxoid, UK), as previously described (De Gheldre et al., 2003; Moremi et al., 2014). *K. pneumoniae* resistant to carbapenems and identified by selective chromogenic medium chromID CARBA (bioMérieux, Florence, Italy) were indicated as *K. pneumoniae* producing carbapenemases (KPC). Methicillin-resistant *Staphylococcus aureus* (MRSA) were those strains that were methicillin resistant on susceptibility testing and producing the penicillin-binding protein (PBP) after confirmation by PBP2′ test kit latex agglutination assay (OXOID Ltd, Basingstoke, UK). Vancomycin-resistant enterococci (VRE) are identified by susceptibility testing and confirmed by selective chromogenic medium chromID VRE (bioMérieux, Florence, Italy). Antibiotic susceptibility was defined by the Minimum Inhibitory Concentration (MIC) interpretative criteria recommended by the European Committee on Antimicrobial Susceptibility Testing (EUCAST–Clinical Breakpoint Table v 5.0). Species numbers and features of the strains tested are reported in Table 1. Isolates were stored at −70°C in CRYOBANK tubes (Copan Italia spa) and grown overnight at 37°C on specific agar plate prior to testing.

**Table 1:** **Characteristics of clinical isolates used in this study**.

**Bacterial species**	**Clinical isolates**	**Phenotype**	**Site of isolation**
**GRAM-NEGATIVE BACTERIA**			
*P. aeruginosa*	9	MDR (2)	CA-BI (2)
		MDR (1)	Chronic Ulcer (2)
		–	Wound (1)
		–	Urine (1)
		–	Respiratory (3)
*K. pneumoniae*	8	KPC (1)	CVC (1)
		–	Blood (2)
		–	Wound (1)
		ESBL (1)	CA-UTI (2)
		–	Respiratory (2)
*R. mannitolilytica*	8	–	Blood (8)
**GRAM-POSITIVE BACTERIA**			
*S. aureus*	10	MRSA (1)	CVC (1)
		MRSA (3)	Chronic Ulcer (6)
		–	Skin (2)
		–	Respiratory (1)
*S. epidermidis*	8	–	Blood (6)
		–	Wound (2)
Other Gram+	9	–	Blood (2)
		VRE (1)	Wound (6)
		–	Urine (1)

Multidrug resistant (MDR), extended spectrum beta-lactamase (ESBL) K. pneumoniae producing carbapenemases (KPC), methicillin-resistant S. aureus, vancomycin-resistant enterococci (VRE). Catheter Associated–Urinary Tract Infection (CA-UTI), Central Venous Catheter (CVC), Catheter Associated Bloodstream Infection (CA-BI).

### Clinical BioFilm Ring Test (cBRT) protocol

For cBRT experiments, a fresh overnight culture of bacteria grown on agar plate was used to inoculate, by a sterile inoculating loop, 2 mL of 0.45% saline solution (AirLife, Carefusion, CA, USA) to the equivalent of 1.0 ± 0.3 McFarland (McF) turbidity standard and thoroughly mixed. 96-well polystyrene plate was inoculated with 200 μL/well of bacteria suspension. The test was performed using toner solution (TON004) (Biofilm Control, Saint Beauzire, France) containing magnetic beads 1% (v/v) mixed in Brain Heart Infusion medium (BHI, Difco, Detroit, MI, USA). Next, a set of 10-fold serial dilutions, from 1 × 10^−1^ to 1 × 10^−6^ were done in a volume of 200 μL BHI/TON mix.

One or more laboratory strains were included in each plate as standard reference and quality control. A well containing the BHI/TON mix without microbial cells was used as negative control in each experiment.

After 5 h of incubation at 37°C without shaking (static culture), wells were covered with a few drops of contrast liquid (inert opaque oil used) placed for 1 min on the block carrying 96 mini-magnets (Block test) and scanned with a specifically designed plate reader (Pack BIOFILM, Biofilm Control, Saint Beauzire, France). The adhesion strength of each strain was expressed as BioFilm Index (BFI), which was calculated by dedicated software according to Chavant et al. (2007). The values of BFI were used to measure the biofilm-forming potential (BP), the latter being a calculation specifically developed for this test, by using the formula: BP = [1−(BFI sample/average BFI of negative control)] for each well. The cut-off (BFIc) value was defined as three standard deviations above the mean of the negative control wells (BFI = 18.75 ± 0.32). Values of BP above two times the cut-off were considered to be significant biofilm formers (2BFIc ≥ 0.53). Thus, the last dilution above 2BFIc identifies the ability of the microorganism to form biofilm. Accordingly, microorganisms were classified into the following categories: poor biofilm-producer (BP < 2BFIc at 1 × 10^−1^ McF), weak (BP > 2BFIc at 1 × 10^−1^ and/or 1 × 10^−2^ McF), moderate (BP > 2BFIc at 1 × 10^−3^ and/or 1 × 10^−4^ McF), and high biofilm producer (BP > 2BFIc at 1 × 10^−5^ and/or 1 × 10^−6^ McF). Each microbial culture was analyzed in duplicate and experiments were repeated at least three times for each strain to assess repeatability, accuracy, and precision of the assay. Values were considered valid when the standard deviation between duplicates did not exceeded 8%. Replicates showed a complete categorical accordance within their classification.

### Assessment of bacterial growth and adherence

To further verify the quality of the inoculum and bacterial growth, the fraction of planktonic and adhering cells was determined using an inoculum of 10^5^ CFU/mL, from a fresh overnight culture of bacteria grown on agar plate, and diluted in 200 μL of BHI medium. Bacterial cells were incubated for 5 h at 37°C in a sterile 96-well polystyrene plates without shaking. The culture supernatants were aspirated to collect the planktonic cells. Adherent cells were first washed twice in saline solution and subsequently detached by scraping the bottom of the wells with a pipet tip and resuspended in 200 μL of sterile distilled water. The cell suspensions from planktonic and adherent cells were serially diluted and 5 μL spotted on agar plates. The mean generation time or doubling time (g) was calculated using the following equation: log_10_ N_t_ = log_10_ N_0_ + g log_10_2 where N_t_ is the number of cells after 5 h of incubation and N_0_ is the number of cells at time zero. The rate of adherence was calculated as the ratio between adherent cells/planktonic cells doubling times (adherence index). All assays were performed in triplicate and repeated in three different experiments.

### Evaluation of the biofilm formation with crystal violet assay

Sterile 96-well polystyrene plates were inoculated with 200 μL of an initial bacterial suspension (10^5^ CFU/ml) in BHI medium and incubated at 37°C for 24 and 48 h without shaking. Each strain was evaluated in triplicate. Medium was removed from the wells, which were washed three times with 200 μL sterile distilled water. The plates were air-dried for 45 min and the adherent cells stained with 200 μL of 0.1% crystal violet solution. After 20 min, the dye was removed and the wells washed four times with 300 μL of sterile distilled water to remove excess stain. The dye incorporated by the cells forming biofilm was dissolved with 200 μL of ethanol/acetone, 80/20% and the absorbance of each well was measured spectrophotometrically at 570 nm (OD570) by using the automated PhD™ lx System (Bio-Rad Laboratories, Hercules, CA, USA).

For comparative analysis the OD570 values were used to classify semi-quantitatively biofilm production for the bacterial strains according to the method described in Stepanovic et al. (2000). Briefly, the cut-off OD (ODc) was defined as three standard deviations above the mean OD of the negative control and strains were classified as follows: OD < ODc = poor biofilm producer; ODc < OD < 2 × ODc = weak biofilm producer; 2 × ODc < OD < 4 × ODc = moderate biofilm producer; and OD > 4 × ODc = high biofilm producer.

All assays were performed in triplicate, with reference strains and clinical isolates, and tested on three different experiments.

### Statistical methods

The κ coefficient test was used to determine the agreement between the results obtained with the cBRT assay and CV. Strength of agreement was calculated according to Altman (Altman, 1991): κ = 0.81–1, very good; κ = 0.61–0.80, good; κ = 0.41–0.60, moderate; κ = 0.21–0.40, fair; κ ≤ 0.20, poor. The results obtained with both procedures were further compared using the McNemar's test. *P*-values of < 0.05 were considered significant.

## Results

### Measurement of bacterial inoculum and fraction of planktonic and adhering cells

Since the initial cell concentration may affect biofilm formation and consequently the reliability of the test, the accuracy of the initial inoculum for the different microorganisms, including mucoid or non-mucoid strains, was determined with a densitometer, and further confirmed by colony-forming unit (CFU) counts (Welch et al., 2012).

The data reported in Table 2, show that 1 McF unit ranged from 0.6 × 10^9^ ± 2.6 × 10^8^ CFU/mL for the *R. mannitolilytica* to 1.4 × 10^9^ ± 7.2 × 10^8^ CFU/mL for *P. aeruginosa*, respectively. The average value of CFU/mL for the planktonic fraction of different bacterial strains grown to 1 McF corresponded to 1.0 × 10^9^ ± 3.6 × 10^8^, consistent with the range of previously reported values (Eng et al., 1984; Bhagunde et al., 2010). Hence, the CFU counts proved that the McF standard is accurate and reproducible, regardless of the bacterial species or microbial phenotypes, ensuring that no substantial differences exist in the initial inoculum when cell concentration is measured by densitometry.

**Table 2:** **Corresponding CFU/mL to 1McFarland for the bacteria used to inoculate the cBRT assay and relative fraction of planktonic and adherent cells**.

**Microbial strains**	**Initial inoculum CFU/mL**
*P. aeruginosa*	1.4 × 10^9^ ± 7.2 × 10^8^
*K. pneumonia*	0.9 × 10^9^ ± 7.9 × 10^8^
*R. mannitolilytica*	0.6 × 10^9^ ± 2.6 × 10^8^
*S. aureus*	1.3 × 10^9^ ± 8.4 × 10^8^
*S. epidermidis*	1.1 × 10^9^ ± 7.1 × 10^8^
Other Gram+	1.1 × 10^9^ ± 8,7 × 10^8^
Average	1.0 × 10^9^ ± 3.2 × 10^8^

The values represent mean CFU/mL ± SD of two replicates.

Subsequently, the growth rate for the 52 clinical isolates and for the 11 laboratory strains was determined by measuring the number of planktonic and adhering cells, respectively (Table 3). This test was performed to evaluate the growth ability of different bacterial strains in the experimental culture conditions. For this assessment, the initial inoculum was of 1.0 × 10^5^ CFU/mL. The results, summarized in Table 3, showed that all the strains had a similar growth rate. In fact, the lower number of planktonic cells was found with *P. aeruginosa* (1.2 × 10^7^ ± 8.8 × 10^6^ CFU/mL) while the higher number was found with *S. aureus* (3.3 × 10^7^ ± 7.5 × 10^6^ CFU/mL) corresponding to a doubling time of 43.5 and 35.9 min., respectively. On the other hand, the fraction of adhering cells varied from 3.8 × 10^5^ ± 2.9 × 10^5^ CFU/mL for *R. mannitolilytica* to 2.5 × 10^6^ ± 1.6 × 10^6^ CFU/mL for the group of the Other Gram+ strains, with a doubling time of 155.9 and 65.6 min., respectively. These results show that after 5 h of incubation all the strains were able to adhere to the bottom of the microtiter-plate with no significant differences between Gram-positive and Gram-negative bacteria. The analysis of the adherence index (i.e., the ratio between the doubling time for the adherent fraction vs. the doubling time of the planktonic fraction), which provides a measurement of adherent cell production by different strains (Table 3), showed that the most efficient Gram-negative species was *P. aeruginosa*, which produced one adherent cell every 1.77 planktonic cells, while the most efficient among the Gram-positive bacteria was *S. epidermidis*, with an adherence index of 1.78 (Table 3). These results proved that variations in biofilm production were related to the specific ability of the different bacterial strains to entrap magnetic nanoparticle within the newly formed biofilm matrix and not the result of defective growth capabilities.

**Table 3:** **Measurement of planktonic and adhering cell fractions for the 52 clinical isolates and the 11 laboratory strains**.

**Microbial strains**	**Planktonic CFU/mL**	**Planktonic doubling time (min)**	**Adherent CFU/mL**	**Adherent doubling time (min)**	AI (^*^)
*P. aeruginosa*	1.2 × 10^7^ ± 8.8 × 10^6^	43.5	1.5 × 10^6^ ± 1.5 × 10^6^	77.0	1.77
*K. pneumoniae*	3.2 × 10^7^ ±7.5 × 10^6^	36.1	1.6 × 10^6^ ± 1.3 × 10^6^	75.6	2.09
*R. mannitolilytica*	2.5 × 10^7^ ± 8.3 × 10^6^	37.7	3.8 × 10^5^ ± 2.9 × 10^5^	155.9	4.13
*S. aureus*	3.3 × 10^7^ ±1.5 × 10^7^	35.9	2.2 × 10^6^ ± 1.5 × 10^6^	67.6	1.88
*S. epidermidis*	2.1 × 10^7^ ± 1.2 × 10^7^	38.7	2.0 × 10^6^ ± 1.3 × 10^6^	69.1	1.78
Other Gram+	2.9 × 10^7^ ± 3.3 × 10^7^	36.6	2.4 × 10^6^ ± 1.5 × 10^6^	65.6	1.79
Average	2.5 × 10^7^ ± 9.7 × 10^6^	37.6	1.7 × 10^6^ ± 7.4 × 10^5^	73.5	1.97

(*) Adherence index (AI) was calculated as the ratio between the doubling time for the adherent fraction vs. the doubling time of the planktonik fraction.

### Assessment of biofilm production in gram-negative and gram-positive bacteria

Among the 12 *P. aeruginosa* strains tested, three were laboratory strains with known ability to form biofilm. These included Pa47085 (Moderate) (Schaber et al., 2007), Pa14 (Weak) (Rahme et al., 1995), and Pa9027 (High) (Stapleton et al., 1993), respectively, while the remaining 9 *P. aeruginosa* strains were clinical isolates collected from hospitalized patients (Table 1).

**Figure 1:**
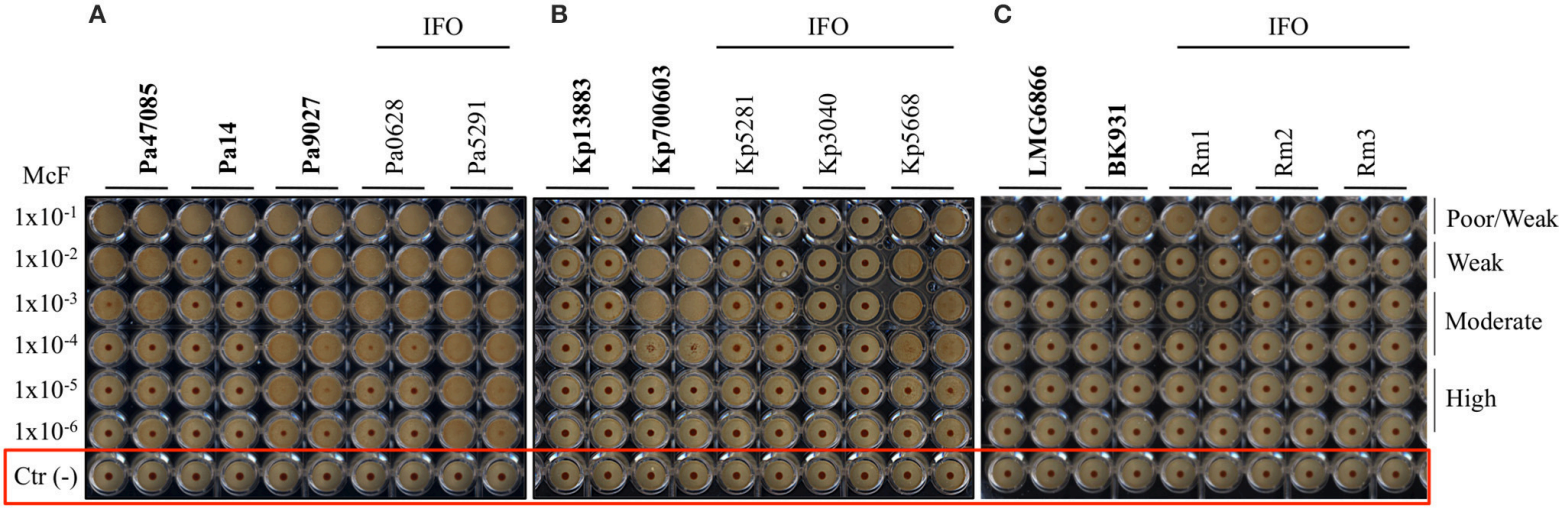
**Biofilm formation on 96 well polystyrene plates for the Gram-negative bacteria after 5 h of incubation at 37°C: (A)** *P. aeruginosa* (Pa), **(B)** *K. pneumoniae* (Kp), and **(C)** *R. mannitolilytica* isolated from the IFO hospital. Images were obtained after magnetization of the plates on the Block Test and scanning with the Plate Reader. The reference laboratory strains are indicated in bold. Negative controls with only BHI medium and magnetic microparticles are circled in red.

After 5 h of incubation, the reference strain Pa14 immobilized the magnetic beads only at the highest cell concentration (McF = 1 × 10^−2^), thus suggesting low biofilm production ability (Figure 1A). Likewise, Pa14 was classified as weak biofilm producer (Table 4A). Conversely, Pa47085 was found to adhere more readily to the surface of the wells blocking the magnetic beads at McF = 1 × 10^−3^, thus identifying this strain as good biofilm producer. Similarly, Pa9027 was confirmed as a high biofilm producer at low cell concentration (McF = 1 × 10^−6^). These results are consistent with those previously reported for these laboratory strains (Stapleton et al., 1993; Rahme et al., 1995; Schaber et al., 2007).

**Table 4:** **Plates analysis obtained with the cBRT protocol for (A) *P. aeruginosa*, (B) *K. pneumoniae* and (C) *R. mannitolilytica***.

**(A) McF**	**Pa47085**	**Pa14**	**Pa9027**	**Pa6020-IFO**	**Pa0115-IFO**	**Pa3019-IFO**	**Pa5797-IFO**	**Pa0628-IFO**	**Pa0629-IFO**	**Pa5252-IFO**	**Pa0118-IFO**	**Pa5291-IFO**		
1x10^−1^	0.97	0.98	0.99	0.98	0.90	0.97	0.99	1.00	1.00	0.97	0.98	1.00	Poor/Weak	
1x10^−2^	0.97	0.72	0.99	0.96	0.33	0.99	1.00	1.00	1.00	0.95	0.97	1.00	Weak	
1x10^−3^	0.96	0.01	0.95	0.92	0.12	0.98	0.99	0.98	0.98	0.98	0.93	1.00	Moderate	
1x10^−4^	0.01	−0.02	0.97	0.74	0.06	0.64	0.87	0.58	0.96	0.96	0.89	1.00	Moderate	
1x10^−5^	0.03	0.03	0.96	0.57	0.07	−0.02	0.53	0.07	0.87	0.92	0.78	1.00	High	
1x10^−6^	0.05	0.05	0.52	0.39	0.08	0.00	0.49	−0.01	0.71	0.87	0.56	0.93	High	
	1	2	3	4	5	6	7	8	9	10	11	12		
**(B) McF**	**Kp13883**	**Kp700603**	**Kp5553-IFO**	**Kp5776-IFO**	**Kp5668-IFO**	**Kp5281-IFO**	**Kp3040-IFO**	**Kp0068-IFO**	**Kp5656-IFO**	**Kp5783-IFO**				
1x10^−1^	0.07	0.98	0.92	0.86	1.00	0.01	0.04	1.00	−0.01	0.15	Poor/Weak			
1x10^−2^	0.08	0.94	0.91	0.81	0.94	0.01	0.04	1.00	0.00	0.05	Weak			
1x10^−3^	0.03	0.82	0.84	0.76	0.95	−0.02	0.04	0.99	−0.01	0.00	Moderate			
1x10^−4^	0.07	0.22	0.37	0.26	0.92	0.01	0.06	0.75	0.04	0.02	Moderate			
1x10^−5^	−0.01	−0.03	0.05	0.00	0.25	0.02	0.00	0.01	0.04	−0.01	High			
1x10^−6^	0.00	0.02	0.02	0.02	0.11	0.04	0.01	−0.05	0.03	0.01	High			
	1	2	3	4	5	6	7	8	9	10				
**(C) McF**	**LMG 6866**	**BK931**	**Rm1-IFO**	**Rm2-IFO**	**Rm3-IFO**	**Rm4-IFO**	**Rm5-IFO**	**Rm6-IFO**	**Rm7-IFO**	**Rm8-IFO**				
1x10^−1^	0.88	0.66	0.88	0.99	0.59	0.57	0.91	0.51	0.57	0.94	Poor/Weak		Legend	
1x10^−2^	0.26	0.14	0.17	0.61	0.13	0.11	0.16	0.12	0.15	0.54	Weak		1	
1x10^−3^	0.09	0.03	0.10	0.13	0.05	0.07	0.07	0.10	0.09	0.15	Moderate		0.75	
1x10^−4^	0.00	0.04	0.07	0.01	0.11	0.12	0.02	0.10	0.10	0.04	Moderate		0.53	Cut-off
1x10^−5^	0.02	0.06	0.06	0.10	0.09	0.12	0.02	0.09	0.10	0.10	High		0.25	
1x10^−6^	0.03	0.10	0.05	0.03	0.09	0.13	0.01	0.09	0.11	0.09	High		0	
	1	2	3	4	5	6	7	8	9	10				

Values reported in the table refer to biofilm-forming potential (BP).

Among the clinical isolates, six strains were found to be high biofilm producers. In particular, these included all the three MDR strains, two deriving from patients with catheter-associated bloodstream infections (Pa6020-IFO, Pa5252-IFO) and one from a chronic ulcer (Pa5797-IFO), and strains isolated from a bronchoalveolar lavage of a patient with Cystic Fibrosis (Pa0629-IFO), from the pleural fluid (Pa5291-IFO) and from a patient with an infected wound (Pa0118-IFO). Two strains derived from a chronic ulcer (Pa3019-IFO) and from the bronchoalveolar lavage from a Cystic Fibrosis patient (Pa0628-IFO), were classified as moderate biofilm producers. The only weak biofilm producer was a mucoid strain isolated from a urinary infection (Pa0115-IFO).

The test was then used to assess the ability of *K. pneumonia* to produce biofilm. The results, summarized in Figure 1B and in Table 4B, showed that the reference strains Kp13883 and Kp700603 were poor and moderate biofilm producer, respectively, confirming previous reports (Naparstek et al., 2014). Regarding the clinical strains, Kp0068-IFO (from a urinary infection), Kp5553-IFO (an ESBL strain from a urinary catheter), Kp5668-IFO (a KPC strain from a central venous catheter) and Kp5776-IFO (from pleural fluid) were found to be moderate biofilm producers. Conversely, the strains isolated from blood cultures (Kp5656-IFO, Kp5281-IFO, Kp5783-IFO, Kp3040-IFO), but not deriving from catheter-related bloodstream infections, showed a mucoid phenotype and were found to be poor biofilm producer. These data are consistent with previous reports indicating a higher frequency of biofilm forming strains among *K. pneumoniae* isolated from non-fluid physiologic environments, and may contribute to explain the difficulty at eradicating these infections once they are established in solid tissues (Sanchez et al., 2013). From the restricted number of clinical isolates analyzed emerged a relatively high incidence of poor biofilm producer strains. Specifically, we observed that only 50% of the *K. pneumoniae* included in this study were able to produce biofilm. This value is in agreement with previous *in vitro* studies demonstrating that only 40% of *K. pneumoniae* isolated from different materials, were able to produce biofilm (Yang and Zhang, 2008).

The *R. mannitolilytica* strains herein analyzed were isogenic clinical isolates from an outbreak occurred in the Oncology ward in our hospital in 2014. Two reference strains, *R. mannitolilytica* LMG 6866 (De Baere et al., 2001), and *R. mannitolilytica* BK931 (Marroni et al., 2003) were assessed for comparison, although their biofilm production ability was not known. Results summarized in Figure 1C and Table 4C revealed that the reference strains LMG 6866 and BK931 were weak biofilm producers. Accordingly, also the 8 *R. mannitolilytica* strains, isolated from blood culture, were poor/weak biofilm producers.

**Figure 2:**
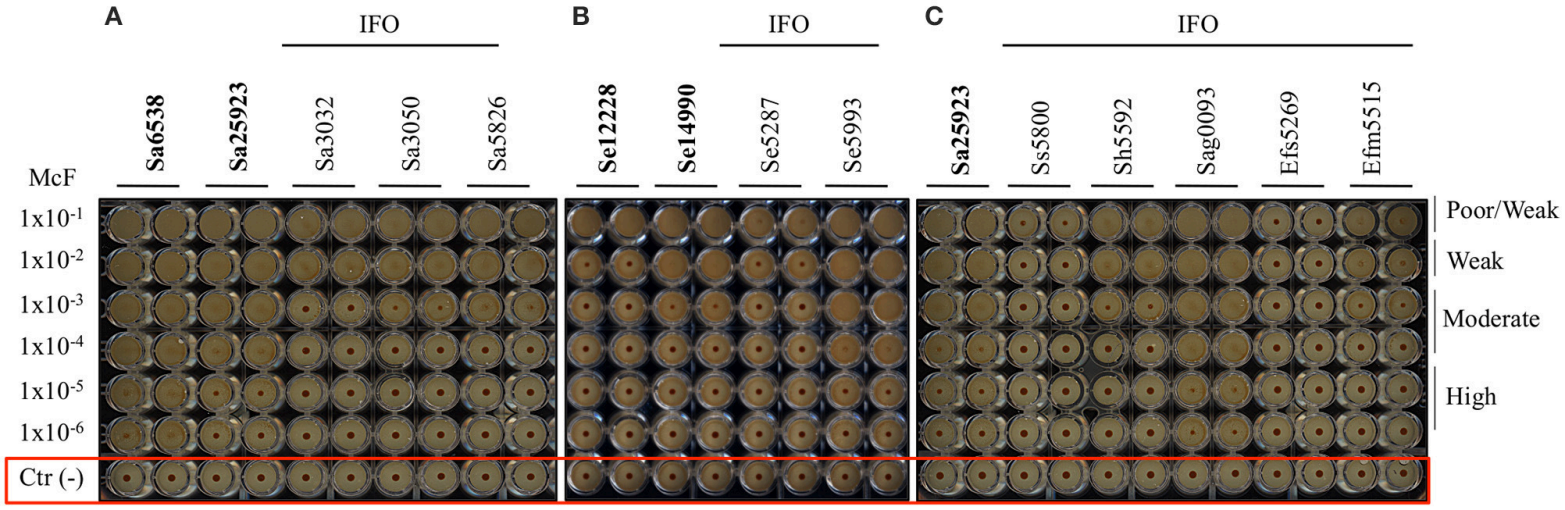
**Biofilm formation on 96 well polystyrene plates for the Gram-positive bacteria after 5 h of incubation at 37°C. (A)** *S. aureus* (Sa), **(B)** *S. epidermidis* (Se), and **(C)** different Gram-positive bacteria isolated from the IFO hospital. Images were obtained after magnetization of the plates on the Block Test and scanning with the Plate Reader. The reference laboratory strains are indicated in bold. Negative controls with only BHI medium and magnetic microparticles are circled in red.

The ability to form biofilm was then explored in different Gram-positive bacteria. As first, different strains of *Staphylococcus aureus* were assessed. According to previous reports (Luppens et al., 2002; Croes et al., 2009; Latimer et al., 2012) the reference strains Sa6538 and Sa25923, were found to be strong biofilm producer (Figure 2A, Table 5A). Among the clinical isolates, five strains were found to be moderate biofilm producers (Sa3074-IFO, Sa3050-IFO, Sa0186-IFO, Sa5674-IFO, and Sa5826-IFO) and three strains, isolated from patients with chronic ulcers, were classified as high biofilm producers (Sa3146-IFO, Sa3079-IFO, and Sa3065-IFO). Within the four MRSA strains tested, two were moderate (Sa0186-IFO, Sa5826-IFO) and two were high biofilm producers, (Sa3146-IFO, and Sa3065-IFO), respectively. The only two weak biofilm producer strains were isolated from children with mild atopic dermatitis (Sa3032-IFO and Sa0073-IFO). None of the strains analyzed were found to be poor biofilm producer. These results are in agreement with previous reports describing the ability of *S. aureus* to produce biofilm (Otto, 2008; Periasamy et al., 2012).

**Table 5:** **Plates analysis obtained with the cBRT protocol for (A) *S. aureus* (B) *S. epidermidis* and (C) Various Gram-positive**.

**(A) McF**	**Sa6538**	**Sa25923**	**Sa3074-IFO**	**Sa3032-IFO**	**Sa3050-IFO**	**Sa0073-IFO**	**Sa0186-IFO**	**Sa5674-IFO**	**Sa5826-IFO**	**Sa3146-IFO**	**Sa3079-IFO**	**Sa3065-IFO**		
1x10^−1^	1.00	1.00	1.00	1.00	1.00	1.00	1.00	1.00	1.00	1.00	1.00	1.00	Poor/Weak	
1x10^−2^	0.99	0.99	0.98	0.99	0.94	1.00	1.00	0.99	0.99	0.98	0.99	0.99	Weak	
1x10^−3^	0.95	0.99	0.92	0.09	0.84	0.44	0.98	0.99	0.98	0.98	0.99	0.97	Moderate	
1x10^−4^	0.94	0.94	0.11	0.10	0.08	0.04	0.56	0.45	0.33	0.98	0.97	0.98	Moderate	
1x10^−5^	0.90	0.54	0.12	0.03	0.10	0.04	0.05	0.02	0.02	0.93	0.74	0.99	High	
1x10^−6^	0.83	0.01	0.03	0.04	0.05	0.03	0.00	0.03	−0.01	0.70	0.64	0.80	High	
	1	2	3	4	5	6	7	8	9	10	11	12		
**(B) McF**	**Se12228**	**Se14990**	**Se5287-IFO**	**Se5669-IFO**	**Se5899-IFO**	**Se1501-IFO**	**Se5934-IFO**	**Se5752-IFO**	**Se5845-IFO**	**Se5993-IFO**				
1x10^−1^	0.99	1.00	0.94	0.97	0.99	0.98	0.97	0.99	0.99	1.00	Poor/Weak			
1x10^−2^	0.59	0.99	0.62	0.98	0.99	0.91	0.84	0.99	0.99	0.98	Weak			
1x10^−3^	0.29	0.81	0.27	0.28	0.64	0.52	0.45	0.46	0.34	0.98	Moderate			
1x10^−4^	0.20	0.27	0.20	0.00	0.05	0.20	0.16	0.12	0.05	0.87	Moderate			
1x10^−5^	0.13	0.28	0.13	0.01	0.03	0.02	0.03	0.01	0.02	0.44	High			
1x10^−6^	0.01	0.02	0.03	0.06	0.02	0.02	0.05	0.03	0.05	0.01	High			
	1	2	3	4	5	6	7	8	9	10				
**(C) McF**	**Sa25923**	**Ss5800-IFO**	**Sh5529-IFO**	**Sh5592-IFO**	**Sag0140-IFO**	**Sag0093-IFO**	**Efm5304-IFO**	**Efm5515-IFO**	**Efs0044-IFO**	**Efs5269-IFO**				
1x10^−1^	1.00	0.69	0.86	1.00	0.93	1.00	0.62	0.94	0.98	0.17	Poor/Weak		Legend	
1x10^−2^	0.99	0.21	0.83	0.90	0.97	1.00	0.48	0.83	0.97	0.09	Weak		1	
1x10^−3^	0.94	0.13	0.25	0.64	0.91	0.96	0.12	0.62	0.95	0.07	Moderate		0.75	
1x10^−4^	0.91	−0.06	0.07	0.12	0.87	0.91	−0.02	0.15	0.39	0.06	Moderate		0.53	Cut-off
1x10^−5^	0.56	0.14	0.06	0.01	0.22	0.81	−0.03	0.05	−0.04	0.05	High		0.25	
1x10^−6^	0.02	0.04	0.04	0.03	0.24	0.62	0.00	0.06	0.03	0.02	High		0	
	1	2	3	4	5	6	7	8	9	10				

Values reported in the table refer to biofilm-forming potential (BP).

The assessment of biofilm production by *S. epidermidis*, revealed that the reference strain Se12228 (Zhang et al., 2003) and Se14990 (Stepanović et al., 2003) were weak and moderate biofilm producers, respectively (Figure 2B, Table 5B). This result is consistent with the previous data (Stepanović et al., 2003; Zhang et al., 2003). The analysis of clinical isolates revealed that 5 out of 6 strains derived from blood cultures (Se5287-IFO, Se5669-IFO, Se5934-IFO, Se5752-IFO, and Se5845-IFO) were weak biofilm producers. Whereas, one strain isolated from a catheter-associated bloodstream infection (Se5993-IFO) was found to be a moderate biofilm producer. The other two *S. epidermidis* strains, isolated from wounds were both classified as moderate biofilm producers.

Other Gram-positive bacteria, recognized as important nosocomial pathogens, were analyzed. These included the *Staphylococcus haemolyticus, Streptococcus sanguinis, Streptococcus agalactiae, Enterococcus faecium*, and *Enterococcus faecalis.* In these experiments the *S. aureus* strain Sa25923 was used as reference strain. The results indicated that the *S. agalactiae* strains (Sag0140-IFO and Sag0093-IFO), both isolated from infected ulcers, were moderate/high biofilm producers (Figure 2C, Table 5C). The *E. faecium* (Efm5304-IFO) was a weak biofilm producer, whereas the VRE strain (Efm5515-IFO) was found to be a moderate biofilm producer.

### Species-specific distribution analysis of the biofilm production phenotype

**Figure 3:**
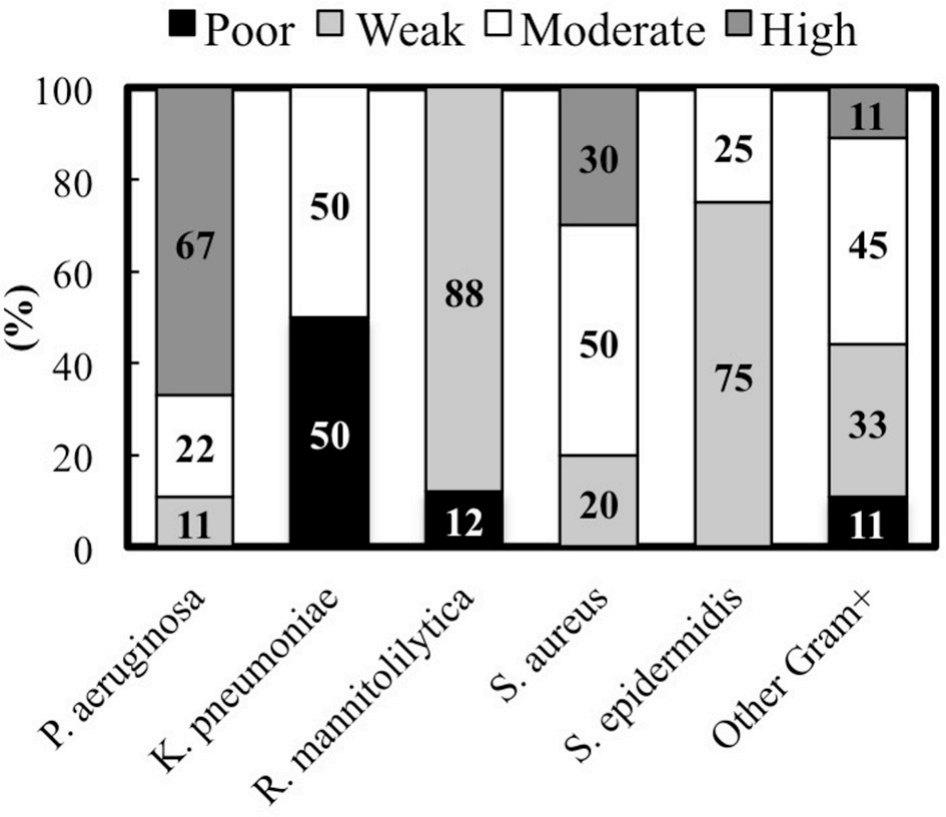
**Percentage of clinical isolates from each species classified according to the biofilm production measured by the cBRT**.

The analysis of the biofilm production according to the different bacterial species indicates that among the Gram-negative bacteria, *P. aeruginosa* showed the most consistent biofilm producer phenotype. In fact, 6 strains (67%) were high biofilm producers, 2 (22%) were moderate and 1 (11%) was a weak biofilm producer, respectively (Figure 3). Conversely, 4 *K. pneumoniae* isolates, which account for 50% of the total, were moderate biofilm producers while the remaining 4 strains were found to be poor biofilm producing bacteria. Interestingly, both the clinical isolates and the laboratory strains of *R. mannitolilytica*, had the weakest biofilm production ability. Among the Gram-positive bacteria, 3 (20%) strains of *S. aureus* were high biofilm producers, 5 (50%) were moderate, and 2 were found to be weak biofilm producers (20%). Within the *S. epidermidis* group of clinical isolates, 6 strains had a weak biofilm producing phenotype (75%) whereas the other 2 (25%) strains were moderate biofilm producers.

Overall, the analysis of the entire bacterial panel, comprising 52 clinical isolates, revealed that more than 44% (23/52) had a moderate/high biofilm producer phenotype and 85% (44/52) were able to produce biofilm. Among the most efficient biofilm producers, we found 89% of *P. aeruginosa*, and 80% of *S. aureus* strains, respectively (Figure 3).

### Categorical agreement of the cBRT with crystal violet staining

**Figure 4:**
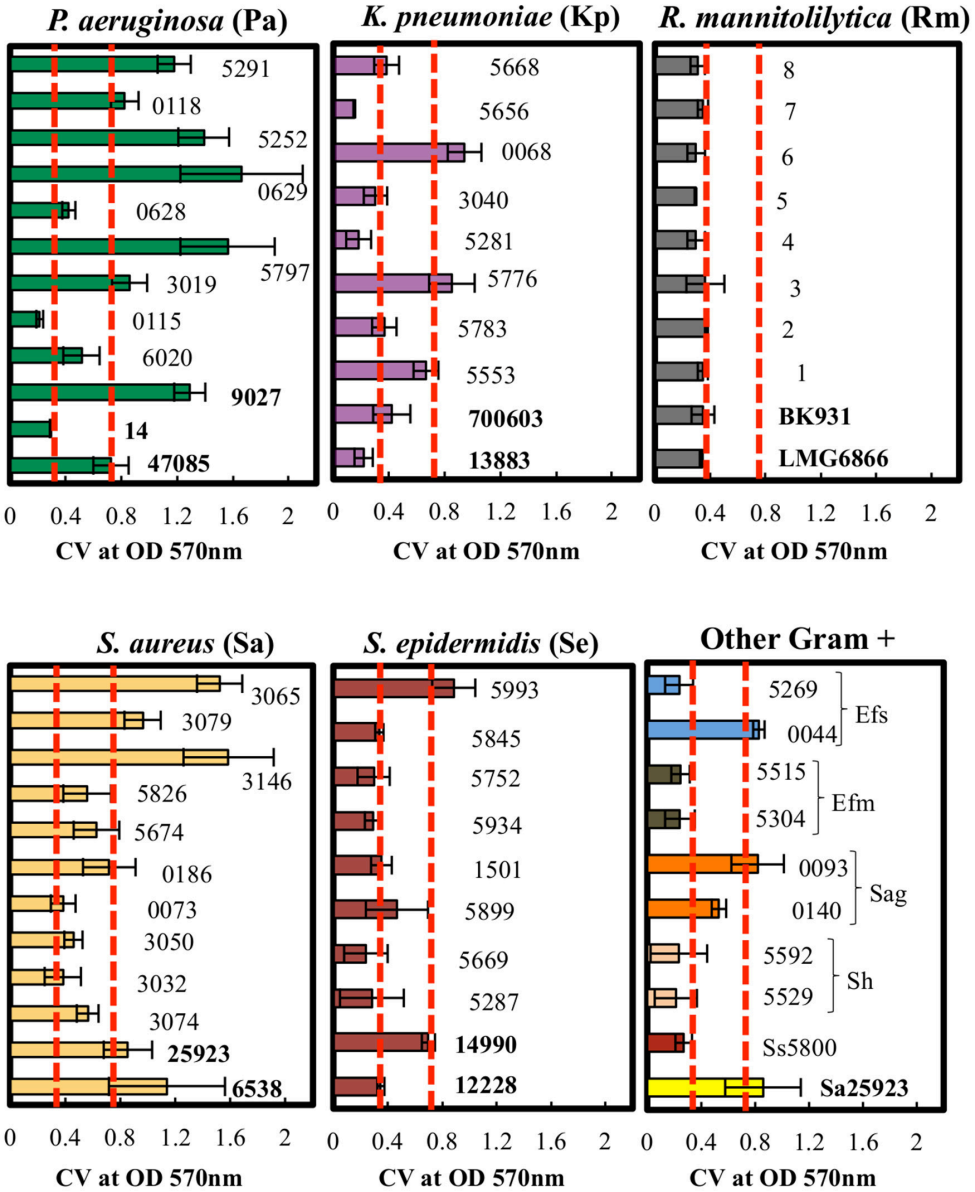
**Quantitative analysis of biofilm formation by CV staining of clinical isolates**. *P. aeruginosa, K. pneumoniae, R. mannitolilytica, S. aureus, S epidermidis*, and other Gram-positive bacteria (Ss, *S. sanguinis;* Sh, *S. haemolyticus*; Sag, *S. agalactiae*; Efm, *E. faecium*; Efs, *E. faecalis*). The isolates were classified according to their cut-off values (Poor = OD570 < 0.18; Weak = 0.18 < OD570 < 0.37; Moderate = 0.37 < OD570 < 0.74; High = OD570 > 0.74). Error bars indicate the standard error. Dashed red lines (- - -) indicate the cut-off values at OD570 < 0.37 and at OD570 < 0.74.

The biofilm production ability of the different clinical isolates was further assessed by the CV assay. The average light absorbance for different biofilm producing bacteria obtained by the CV assay is shown in Figure 4. The results revealed that the repeatability of the CV assay was generally good with only minor differences observed among the replicates.

The results obtained by the CV assay were then compared with the data gathered by the cBRT. A full categorical agreement was defined as the percentage of isolates that were classified in the same category with both methods. A partial categorical agreement was recorded when the same classification was obtained by cBRT and the CV OD570 ± standard deviation (Table 6). Isolates that did not score in the full or partial categorical agreement were considered as being in disagreement.

**Table 6:** **Overall results of adherence for the various bacterial species obtained by the cBRT protocol and CV staining**.

**Bacterial Species**	**cBRT**				**CV staining**				**Categorical agreement (%)**	
	**P**	**W**	**M**	**H**	**P**	**W**	**M**	**H**	**Full**	**Full + Partial**
*P. aeruginosa*	0	2	3	7	0	2	3	7	83	100
*K. pneumonia*	5	0	5	0	1	4	3	2	40	80
*R. mannitolilytica*	1	9	0	0	0	10	0	0	80	100
*S. aureus*	0	2	6	4	0	1	7	4	92	100
*S. epidermidis*	0	7	3	0	0	7	2	1	80	100
Other G+	1	3	4	1	0	6	1	2	60	90

Partial categorical agreement: isolates classified by the cBRT in the same category of the CV OD570 ± standard deviation. P = Poor, W = Weak, M = Moderate, H = High. Full categorical agreement: isolates identified in the same category by both procedures.

Globally, the full categorical agreement for the Gram-negative strains was 68% whereas for the Gram-positive bacteria it was more than 77%, showing an overall agreement in 72.5% of the samples analyzed. The partial categorical agreement was found to be more than 95%, corresponding to 3 misclassifications out of a total of 63 strains analyzed. Importantly, for all the discordant results, classification differed by only one category.

In particular, the full and partial categorical agreement between cBRT and CV assay for *P. aeruginosa* were of 83% and 92%, respectively. For *K. pneumoniae*, two strains were classified as poor biofilm producer by the cBRT (Kp3040-IFO; Kp5783-IFO) and as weak biofilm producers by the CV, while a moderate biofilm producer strain (Kp0068-IFO) as found by cBRT, was classified as high biofilm producer by CV. In these cases the cBRT and CV showed a partial accuracy of 70%. Regarding the *R. mannitolilytica* strains, a 90% agreement was found between cBRT and CV, corresponding to only one misclassification. A complete categorical agreement between tests was observed with both *S. aureus* and *S. epidermidis*, whereas for the other Gram-positive bacteria the agreement was 87%, corresponding to only one misclassification (Ef5515-IFO).

The categorical agreement between cBRT and CV staining was further analyzed by applying the McNemar's test in order to estimate the specific distribution of false positive and false negative cases. The results indicated that, for the discordant identifications, a statistically significant difference exists between tests (*P* = 0.007). Specifically, the CV overestimated biofilm production as compared to the cBRT. This result is not surprising since it could be ascribed to the non-specific staining property of the CV. Indeed, CV is known to bind surface negatively charged molecules, which are present on both the bacteria and the biofilm extracellular matrix (Extremina et al., 2011). This may lead to an overestimation of the actual biofilm producing ability of different strains (Pan et al., 2010; Merritt et al., 2011; Skogman et al., 2012).

The concordance between the cBRT and the CV was statistically measured by the κ coefficient. The results showed a good agreement between tests for the weak (κ = 0.71 ± 0.09; specificity = 94.3%; accuracy = 85.7%), moderate (κ = 0.63 ± 0.11; specificity = 84.8%; accuracy = 84.1%), and high (κ = 0.73 ± 0.10; specificity = 97.9%; accuracy = 90.5%) biofilm producers, while for the poor biofilm producer cells the strength of agreement was moderate (κ = 0.42 ± 0.2; specificity = 91.8%; accuracy = 92.1%). Overall, the strength of agreement between these methods was good (κ = 0.66 ± 0.07), with the cBRT showing high levels of specificity and accuracy (specificity = 92.2%; accuracy = 88.1%).

## Discussion

Microbial biofilm represents a major virulence factor, accounting for a most effective microbial invasiveness and persistence in the environment and in the host. Simple, cost-effective and reliable tests to assess biofilm formation still represent an unmet need for clinical microbiology. In fact, current methods to evaluate biofilm formation are usually time-consuming, costly, and hardly applicable in the clinical setting.

The aim of the present study was to develop a simple and reliable *in vitro* procedure for the determination and quantification of biofilm-production for future clinical applications, based on the BioFilm Ring Test® (BRT) technology. The concept behind the BRT technology is attractive in its simplicity since it is based on the measurement of the ability of bacteria to trap magnetic microparticles within the biofilm matrix. However, the methodological approaches proposed in previous studies posed important limitation for use of BRT for clinical microbiology testing, providing limited information about the characteristics and “strength” of microbial biofilm formation by different microorganisms. In particular, the original protocol proposed by Chavant et al., was based on repeated measurements, performed at different time points, to estimate biofilm formation (Chavant et al., 2007), while, in subsequent studies (Sulaeman et al., 2010; Crémet et al., 2013), single measurements at 2 and 24 h, respectively, were proposed. However, in all these cases, fixed bacterial concentration were used, posing further, important limitations, since, as clearly demonstrated in our study, the analysis of a single cell concentration (at a single time point) is not appropriate to evaluate the strength of biofilm formation. In fact, we found that at high bacterial concentration the majority of microbial strains appear capable of trapping the magnetic beads after 5 h of incubation (and in many cases even after 2 h). However, at low bacterial concentration only the strong biofilm-producing strains are able to prevent the aggregation of the magnetic beads. Moreover, by extending the incubation time for up to 24 h, as described in Crémet et al. (2013), all the strains appear capable to somehow trap the microparticles. All the above limitations can strongly affect the discriminatory power of the test and the reliability of the results.

To overcome these limitations we have introduced substantial modifications to the original protocols. Namely, instead of using different time points to estimate the strength of biofilm formation we introduced a single determination. By reducing the time of incubation to just 5 h we limited the processing time avoiding repeated measurements. However, in place of using a single bacterial concentration we used different dilutions, based on the concept that the fewer concentrations of bacterial cells that inhibit the aggregation of the microparticles in 5 h, the stronger is their ability to produce a biofilm. Conversely, if high bacterial concentrations are not able to prevent the aggregation of the microparticles they are considered low biofilm producers.

In addition, to increase the intra-laboratory reproducibility and the inter-assay control/validation, reference strains with well-defined biofilm production ability have been introduced.

Further, a standard approach for assay measurement was adopted to generate the heat chart using the negative control as cut-off value: BP = [1-(BFI sample/average BFI of negative control)] and the numeric evaluation obtained by the delta BioFilm index (BFI) was substituted with a more intuitive and explicative classification (poor-weak-moderate and high biofilm producers).

The modifications introduced by the cBRT (short time of incubation, absence of extensive handling, internal control/s, standard cut-off calculation and easy interpretation of the results) represent a step beyond the state-of-art as compared to the original methods previously proposed, providing a net improvement in term of standardization of the assay and clarity and comparability of the readouts.

The ability of the cBRT to assess biofilm production was compared with the CV assay, which is a widely used method for biofilm quantification (Christensen et al., 1985; Stepanovic et al., 2000; Peeters et al., 2008). According to the κ coefficient, the overall accordance between tests was satisfactory (κ = 0.66 ± 0.07) with the cBRT showing a higher specificity (92.3%) and accuracy (88.1%) as compared to CV. The full categorical agreement between cBRT and CV was of 72.5%, whereas the partial categorical agreement exceeded 96%, with only two samples misclassified out of 63 strains analyzed. Specifically, the Gram-negative bacteria showed a reduced accordance between tests when compared with the Gram-positives. The full categorical agreement was of 68% for the Gram-negatives and 77% for the Gram-positives, while the partial categorical agreement was of 93.3 and 96.6%, respectively. This apparent discrepancy was mainly accounted by the *K. pneumoniae* strains, which were found to be the bacterial species with the lower categorical agreement between tests (40% full and 80% partial). Indeed, the higher number of discordant results fell within the group of poor/weak biofilm producers. In fact, all these strains showed a mucoid phenotype and this may have affected, at least partially, the sensitivity of the CV test, which is a non-specific colorimetric assay. Since CV stains both live and dead bacteria as well as the biofilm matrix, it can provide only an indirect quantification of biofilm formation, and this may lead to an overestimation of the results due to non-specific staining, particularly when testing mucoid strains (Pan et al., 2010; Merritt et al., 2011; Skogman et al., 2012). Of note, the overproduction of mucus does not play a significant role in bacterial attachment and biofilm matrix formation, although it has been shown to exert protective roles against the host immune response (Stapper et al., 2004; Leid et al., 2005).

Conversely, the cBRT, which is based on the physical ability of the microorganisms to generate a biofilm matrix, which traps magnetic beads (Chavant et al., 2007), provides a direct measurement of the genuine ability of different bacterial strains to produce biofilm. Notably, for all the reference strains, the results gathered by cBRT were in total agreement with the data previously reported in the literature. In addition, the different specificity of the two methods was further confirmed by the McNemar test, which revealed that in those cases in which cBRT and CV showed a disagreement, the CV significantly tended (*P* = 0.007) to overestimate the results. Other important differences between cBRT and CV are related to the feasibility for standardization and the time necessary to complete the assay. With the cBRT plate preparation requires about 30 min plus 5 h of incubation and a few minutes for plate analysis. Conversely, the CV assay requires 24/48 h of incubation, repeated washing steps, and laborious staining procedures (Stepanovic et al., 2000; Djordjevic et al., 2002). The need for extensive handling procedure to perform the CV assay has been indicated as a main cause for large intra- and inter-experimental variations, which lead to often very large standard deviations (Peeters et al., 2008). A limited reproducibility is also one of the main drawbacks presented by other conventional techniques, particularly in high throughput screening (Peeters et al., 2008).

The cBRT assay was tested by assessing different bacterial species, with distinct biofilm-forming phenotypes, including both laboratory strains, and clinical isolates. The measurement of planktonic and adhering cells for all bacterial strains showed that all were able to adhere to the bottom of the wells after 5 h of incubation. Nevertheless, the ability to adhere to a surface does not provide specific information about biofilm production (Donlan, 2002), although the level of extracellular (i.e., biofilm) matrix production has an impact on the adhesion ability of different strains (Vu et al., 2009; Horn et al., 2013; Dertli et al., 2015). The proportion of extracellular matrix in biofilms produced by different strains can vary largely according to the physiological state and nutrient availability, ranging from 50 to 90% of the total organic matter (Vu et al., 2009; Schwartz et al., 2012). Consequently, the medium composition may have a strong impact on the ability of a bacterial strain to form biofilm and this may have important implications for assay standardization. To explore this issue, we tested cBRT also using Tryptic Soy Broth (TSB) as growth medium and we did not observe significant differences with respect to the BHI. We also assessed simple saline solutions (0.45 and 0.90%, respectively), but these media did not support an effective bacterial growth. Thus, the BHI was chosen as the working medium for assay standardization. The use of this medium has been further validated by the results gathered with the 11 reference strains. With the exception of the *R. mannitolilytica*, all the laboratory strains were well characterized in term of their biofilm production ability by different methods, in various conditions and in separate laboratories.

A further issue, dealing with assay standardization, regards the use specific plates. In fact, cBRT standardization was based on the use of polystyrene microtiter plates. However, it is conceivable that different materials might give differing results. Similarly, the cut-off values calculated in the present study should be considered valid exclusively under the standard conditions tested. It is therefore necessary, in the pursuing of an optimal standardization of operating procedures, to further extend the analysis to additional materials and growth media.

From a clinical perspective it is interesting to note that the strains with multiple drug resistances were all found to belong to the moderate/high biofilm producer group. Indeed, as previously shown, MDRs are more frequently associated with high biofilm production (Kwon et al., 2008; Rao et al., 2008; Abidi et al., 2013; Sanchez et al., 2013). The latter appears to represent a key feature that can promote antimicrobial resistance by selecting highly resistant strains exposed to sub-inhibitory antimicrobial concentrations and by providing favorable conditions for gene transfer (Wang et al., 2010). Nevertheless, the relationship between biofilm formation and antibiotic resistance is still a debated issue to researchers. Several studies have reported that sub-lethal dose of certain antibiotics induce biofilm formation (Hoffman et al., 2005; Kaplan, 2011; Mirani and Jamil, 2011) suggesting that biofilm production may represent a common response to different external stressors (Kaplan, 2011). Other reports describe an inverse relationship between biofilm formation and antibiotic resistance. Studies on *Acinetobacter baumannii* contribute to generate uncertainty around this topic since Gurung et al. reported a positive relationship between biofilm formation and antibiotic resistance (Gurung et al., 2013), whereas Perez noticed that strains resistant to meropenem resulted weak biofilm producers (Perez, 2015) and Qi et al. observed that the population exhibiting a more robust biofilm formation likely contained larger proportion of strains sensitive to several antibiotics (Qi et al., 2016). However, these reports are generally based on single-species observations and it is currently unclear whether there is a quantitative correlation between biofilm formation and antibiotic resistance (Qi et al., 2016). In any case, biofilm-embedded microbial cells are more resistant against environmental stress conditions and present an increased tolerance to antimicrobials (Beloin and Ghigo, 2005; Percival et al., 2015). *In vitro* and *in vivo* experiments demonstrated that within a biofilm matrix, cells show a much higher MIC (approximately 10–1000 times) than the same bacterial cells examined in planktonic growth conditions (Høiby et al., 2011; Hengzhuang et al., 2012). The effective antibiotic MIC *in vivo* for eradication of biofilm-embedded microbial cells might be therefore impossible to reach by the administration of antibiotics at doses that appear effective against the planktonic growth fraction, due to the toxicity, and the side effects of the drugs, including limitations imposed by renal and/or hepatic functions.

In very preliminary studies aimed at measuring biofilm production by cBRT and evaluating the effectiveness of antibiotic treatment based on classic drug resistance profiling (antibiogram), data revealed that the antibiotic treatment chosen on the basis of the antibiogram was effective in 3 out of 5 cases of medical devices-related infections caused by weak biofilm producer strains. On the contrary, in 2 out of 5 patients, harboring infections caused by high biofilm producers, the antibiotic treatment chosen on the basis of the antibiogram failed, leading to the removal of the devices. Despite the limited number of cases, these results suggest that a correlation exist between the “strength” of biofilm production and the clinical outcome of the therapeutic intervention based on the antibiogram. Thus, the assessment of the strength of biofilm formation may help identify high-risk infections and may help predict the risk of therapeutic failure, thus providing a key decision-making element in support to an effective therapeutic management of “difficult infections,” such as those associated to medical devices (i.e., starting the antibiotic treatment in the presence of poor/weak biofilm producers or, alternatively, anticipating the removal of the device in the presence of high biofilm producers). In fact, these infections often result in treatment failure and device removal, despite the implementation of apparently appropriate therapeutic strategies (Costerton et al., 2003). In this view, other important applications are foreseen in dentistry, where biofilm is associated with major dental diseases such as dental caries and periodontal disease. In addition to clinical applications, this technology may offer a valuable tool in the food as well as sanitation industry, including water, and air systems.

Thus, the cBRT represents a promising tool for clinical microbiology and may lead to future applications including the possibility for a direct antimicrobial drug profiling of biofilm-producing bacteria, to support most effective therapeutic interventions, as well as the screening of new anti-biofilm agents.

## Author contributions

Conceived and designed the research: ED, LT, and FE. Performed the experiments: ED, CP, GP, MG, and VB. All authors analyzed data. Contributed reagents/materials/analysis tools: FA, FP, CP, TB. Wrote the paper: ED, LT, and FE. All the authors read and approved the final version of the manuscript.

### Conflict of interest statement

The authors declare that the research was conducted in the absence of any commercial or financial relationships that could be construed as a potential conflict of interest. TB is the inventor of the BioFilm Ring Test^®^ and founder of BioFilm Control.
